# What I find difficult in BPBP

**DOI:** 10.1186/1753-6561-9-S3-A13

**Published:** 2015-05-19

**Authors:** Anil Bhatia

**Affiliations:** 1 Joshi Hospital, Pune, Maharashtra, 411004, India

## 

Management of obstetrical palsies is a challenge for a variety of reasons.

Explaining and counseling the parents: There is no doubt that the injury has occurred because of excessive force in delivering the baby. However, this has to be conveyed without blaming either the midwife or the obstetrician. The deficit is usually obvious to the clinician but the parents find it difficult to accept. The concept of nerves and the fact that different nerves are responsible for different functions is fundamental. Very often, ordinary household examples are necessary to make them understand the severity of the injury without, at the same time, frightening them about the future consequences. An honest and detailed discussion is necessary at the outset.

Offering surgery: Extensive palsies are evident and most parents accept surgical treatment readily. The real difficulty is explaining the uncertain and, often, limited possibility of restoration of function.

The majority of patients have suffered lesions of the C5 and C6 roots. There is no doubt that surgery should be offered early if spontaneous recovery is not seen. The rate of progressive improvement is the crux and repeated observations are necessary. The pectoralis major function enables trick movements that can fool the clinician. Incorrect interpretation of the function at the early stages can push us to defer surgery. The consequences of such a conservative attitude are only evident over two-three years when the shoulder function remains poor with development of deformities. Only experience can help arrive at a better evaluation.

Evaluation of nerve stumps and trimming: The primary operation in obstetrical palsies is always systematic exploration of the brachial plexus. The upper trunk is identified with the help of the suprascapular nerve and it is traced proximally. The phrenic nerve is isolated and the nerves are examined at the intervertebral foramina. The branches to the serratus anterior should be looked for and stimulated (under the microscope). The appearance of the sectioned stump needs careful evaluation. The quality of shoulder function restored depends on the number of growing axons directed to the posterior division of the upper trunk. Dissection within the foramina is often difficult. One must reach proximal to the zone of injury to ensure use of a good stump.

Post-operative immobilization: Movements of the head and neck must be prevented. This is particularly true when nerve grafting is done. Older children are, obviously, more active and immobilizing them is a daunting task. The parents are instructed to maintain strict attention throughout the waking hours for a month.

Post-operative therapy: Most children are operated upon at 4-5 months of age. Nerve transfers such as ulnar-biceps or intercostals to musculocutaneous produce contractions within 3-4 months. However, it is difficult to communicate the mechanism of activating the muscle to an 8 months old baby. The parents are instructed to encourage the child to grasp objects in a manner designed to produce biceps action. The child will use the biceps only when it perceives strong contraction of the muscle. This task is even more difficult for intercostals. Often, we can see the biceps contracting when the child cries but active elbow flexion can take two years to appear. Automatically, the use of the hand function is delayed.

Incorporation of the restored function in daily activity: Delay in nerve reconstruction prolongs the period of weak shoulder and elbow functions. As a result, the child cannot reach out for an object and take it to the mouth at the suitable time. This pushes me to offer surgery at an early stage if the shoulder abduction and biceps do not appear by three months.

